# Cervical Sagittal Imbalance after Cervical Laminoplasty in Elderly Patients

**DOI:** 10.1155/2020/8810540

**Published:** 2020-11-30

**Authors:** Hyun Woong Mun, Chang Duk Yuk, Tae Hwan Kim, Moon Soo Park, Seok Woo Kim, Ji Hee Kim, Jun Hyong Ahn, In Bok Chang, Joon Ho Song, Jae Keun Oh

**Affiliations:** ^1^Department of Neurosurgery, Hallym University Sacred Heart Hospital, Anyang, Republic of Korea; ^2^Department of Orthopedics, Hallym University Sacred Heart Hospital, Anyang, Republic of Korea

## Abstract

**Purpose:**

To determine the effect of age on sagittal cervical alignment after cervical laminoplasty procedure so as to identify the group with the greatest degree of variation. *Study Setting*. Single-center retrospective chart review in a tertiary referral hospital. *Outcome Measures*. The **s**agittal vertical axis (SVA) (C2-7), T1 slope, and cervical lordosis.

**Methods:**

We included patients who underwent cervical laminoplasty between 2014 and 2018 and divided 60 consecutive patients into two groups using the cut-off age of 65 years. The Paired *t*-test and Mann-Whitney *U* test were used to compare changes between preoperative radiographic cervical sagittal parameters and those 1 year after surgery.

**Results:**

Mean patient ages in the older and younger groups were 71 years and 52 years, respectively. The difference of C2-7 SVA was greater in the older group.

**Conclusion:**

Postoperative cervical balance can be worse when laminoplasty is performed in elderly patients.

## 1. Introduction

As the elderly population grows, there have appeared more patients who require surgery for cervical compressive myelopathy [1–3]. Laminoplasty is a motion preservation surgical procedure for the treatment of cervical myelopathy and ossification of the posterior longitudinal ligament [4]. Laminoplasty is popular because of its safety and effectiveness, especially when multilevel cervical compression is needed [5–8].

Although there are various benefits of laminoplasty, some studies have demonstrated that the procedure often gives rise to cervical sagittal imbalance attributable to injury of posterior structures which include laminae, articular facets, muscles, and ligaments. Postoperative complications of laminoplasty cause persistent neuropathic arm pain, axial neck pain, and postoperative deterioration of cervical alignment due to the progression of kyphosis [9–12]. There have been some studies of postoperative laminoplasty-related kyphotic deformity suggesting that such changes in alignment could lead to poor surgical results. As the deformity progresses, the compression of the spinal cord could deteriorate, and stresses could cause chronic ischemic changes due to injury of small vessel tributaries of bone [13].

To our best knowledge, no previous study has evaluated the difference of postlaminoplasty cervical sagittal alignment changes between older and younger patients. Therefore, we are aimed at determining the meaningful difference between these groups by measuring the cervical balance parameters.

## 2. Materials and Methods

We predicted that the postoperative changes in cervical sagittal balance will be more severe in elderly patients than in younger patients, and that this difference could be evaluated using sagittal balance parameters. We conducted a retrospective single-center study, including patients who presented to our department for cervical laminoplasty using surgical method both “french door” laminoplasty and “open door” laminoplasty, from 2014 to 2018. We enrolled 98 patients, but excluded patients with tumor, trauma, history of previous cervical spine surgery, and who could not maintain an upright position without assistance. 60 people included and divided them into two groups using age 65 years as the cut-off for two groups even 30 people. We recorded characteristics including age, gender, and diagnosis. Cervical lateral X-ray was obtained using an Infinitt PACS (Infinitt Healthcare Co., Ltd., Seoul, Korea) to measure sagittal balance parameters, including C2-7 sagittal vertical axis (SVA), cervical lordosis (CL), T1 slope, and T1 slope minus cervical lordosis (T1 slope-CL) with accurate position. The follow-up period was set at 1 year to evaluated postoperative changes. We excluded patients who had had previous cervical surgery or cervical vertebral fracture because they were difficult to assess using radiographical parameters.

**Figure 1:**
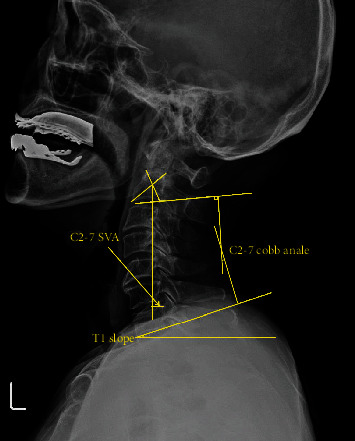
Illustration of cervical sagittal balance. Radiographic measurements. C2-7 cobb angle. C2-C7 SVA. T1 slope.

To evaluate cervical sagittal balance, we measured the following parameters on cervical lateral X-ray: C2-7 SVA, T1 slope, CL, and T1 slope-CL. The C2-7 SVA was defined as the total distance from the plumb line of the pedicle center of the C2 vertebra to the posterior superior corner of the C7 vertebra [14]. The CL was measured using the Cobb angle of C2 lower endplate and C7 lower endplate [15]. T1 slope was defined as the angle between the horizontal plane and T1 upper endplate (Figure 1) [16, 17].

We use SPSS software (version 22.0, SPSS, Chicago, IL, USA) to evaluate postoperative changes based on Paired *T*-test and Mann-Whitney *U* test. *P* values < 0.05 were considered statistically significant.

## 3. Results

We measured the range, mean, and standard deviation of each parameter and characteristics. We are aimed at determining whether elderly patients showed more significant postoperative changes than younger patients. There were 30 patients in the older groups (men, *n* = 18). The mean age was 70.80 ± 5.03 years. The preoperative data were as follows: C2-7 SVA range -0.60 to 5.10 mm, mean 2.09; CL range 2.70 to 31.60°, mean 13.98; T1 slope range 4.90 to 48.60°, mean 26.16; and T1 slope-CL range 2.10 to 27.10°, mean 13.22 (Table 1).

There were 30 patients in the younger group (men, *n* = 20). The mean age was 52.53 ± 7.68 years. The preoperative data were as follows: C2-7 SVA range 0.70 to 4.80 mm, mean 1.97; CL range 5.00 to 33.00°, mean 12.59; T1 slope range 15.00 to 46.00°, mean 25.43; and T1 slope-CL range -1.00 to 29.50°, mean 12.84 (Table 1).

**Figure 2:**
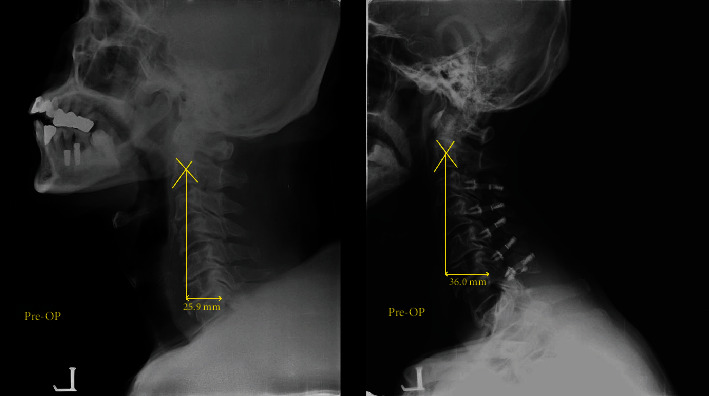
C2-7 SVA increased after cervical laminoplasty (pre-op: 25.9 mm → post-op 36.0 mm).

The postoperative change of C2-7 SVA tended to be greater than preoperative values in both age groups at 1-year follow-up. In the older group, C2-7 SVA changed from 2.09 mm preoperatively to 3.39 mm postoperatively (*P* < 0.01). CL changed from 13.98° to 13.04° (*P* = 0.402), T1 slope changed from 26.16° to 28.84° (*P* = 0.075), and T1 slope-CL changed from 13.22° to 17.19° (*P* = 0.017). In the younger group, C2-7 SVA changed from 2.31 to 2.94 (*P* < 0.01). CL changed from 12.59° to 14. 03° (*P* = 0.234), T1 slope changed from 25.43° to 28.10° (*P* < 0.01), and T1 slope-CL changed from 12.91° to 14.73° (*P* = 0.112) (Figure 2, Table 2).

We also evaluate both groups together. The C2-7 SVA changed from 2.20 mm preoperatively to 3.16 mm postoperatively (*P* < 0.01). CL changed from 13.28° to 13.54° (*P* = 0.758), T1 slope changed from 25.79° to 28.47° (*P* < 0.01), and T1 slope-CL changed from 13.06° to 15.96° (*P* < 0.01) (Table 3).

To compare postoperative changes between the two groups, we used Mann-Whitney *U* test. There was a statistically meaningful result only in postoperative C2-7 SVA minus preoperative C2-7 SVA between older and younger groups (*P* < 0.01). The other parameters such as T1 slope, CL, and T1 slope-CL showed any statistically significant results (Table 4).

## 4. Discussion

Laminoplasty is a better procedure than cervical laminectomy because it maintains cervical alignment and postoperative kyphosis [14, 18]. Nevertheless, there have been several studies reporting that cervical laminoplasty reduced the range of motion and caused cervical malalignment, though these issues remain matters of debate. Because loss of lordotic curve after laminoplasty is a major complication, and many spine surgeons increasingly pay attention to postoperative cervical sagittal alignment because kyphotic change may significantly decrease the quality of life after laminoplasty [19–21].

**Figure 3:**
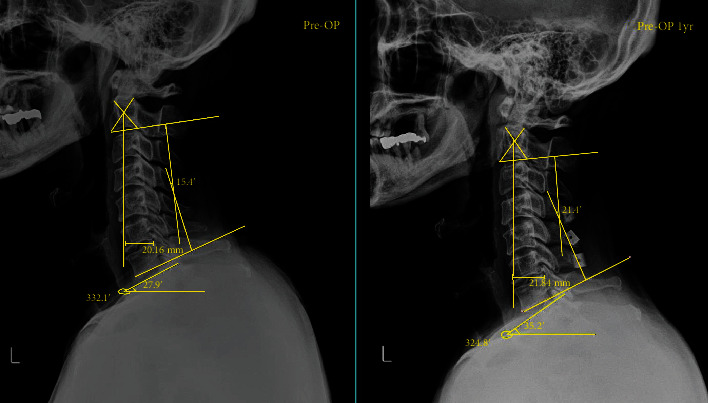
After laminoplasty, T1 slope, cervical lordosis, and C2-7 SVA tend to increase (pre-op T1 slope: 27.9° → post-op 35.2°, pre-op C2-7 cobb angle: 15.4° →21.4°, pre-op C2-7 SVA 20.16 mm →21.84 mm).

There have been many studies to explain the mechanism of postlaminoplasty sagittal imbalance; nevertheless, those mechanisms remain unclear. Lee et al. stated that the T1 slope was a key factor determining cervical spine sagittal balance [22]. In our study, there was a tendency of increased T1 slope in patients who underwent cervical laminoplasty in both age groups. In the case of cervical lordosis, the younger patients showed positive changes, whereas older patients showed the opposite tendency. We assume that there could be a compensatory mechanism in cases of T1 slope increases to maintain sagittal balance as cervical lordosis increases. In the younger patients, a compensatory mechanism effectively increased cervical lordosis, but this did not occur in elderly patients. For this reason, we believe that the C2-7 SVA was significantly greater in older patients than in younger patients (Figure 3).

We also measured postoperative alignment changes after laminoplasty. We hypothesized that age would be a poor prognostic factor in the postoperative outcome in terms of alignment. We found that changes in C2-7 SVA in the elderly group are more prominent than in the younger group. Koshimizu et al. evaluated the effects of sarcopenia on the sagittal alignment of the cervical spine after cervical laminoplasty [23]. As in our results, they showed that C2-7 SVA was greater, and postoperative outcome was worse after cervical laminoplasty in the sarcopenia group [23]. Several muscles, including the cervical multifidus and trapezius muscles that support the cervical spine, are thought to disrupt cervical sagittal balance as the muscle mass decreases with age. This may be why elderly patients in our study had a greater breakdown of sagittal balance after cervical laminoplasty than did the younger patients.

Prior to laminoplasty, surgeons need to advise patients regarding postoperative kyphosis that cause several surgical complications. Regarding decompression of the cervical canal with pathologies such as cervical myelopathy, patients should be carefully selected. Sakai et al. reported that postoperative cervical sagittal alignment tended to deteriorate following laminoplasty, especially in patients with preoperative center-of-gravity-of head-C7 sagittal vertical axis ≥ 40 mm [24]. Especially in elderly patients with large SVA, there must be careful decision-making applied to laminoplasty planning, because of significant C2-7 SVA enlargement. Our data also suggest that surgeons should consider intraoperative technique aimed at reducing postlaminoplasty complications such as kyphotic changes. Several studies have addressed methods of cervical laminoplasty; however, there has been no consensus regarding the superiority of either single-door laminoplasty or double-door laminoplasty. Nevertheless, there is general agreement that a lesser degree of exposure of the semispinalis cervicis muscle gives rise to better preservation of cervical lordosis [19]. The simplest way to reduce complications is to take care to minimize injury to cervical muscles and ligamentous supports of the posterior column, particularly in elderly patients. Furthermore, early rehabilitation is important because the postoperative recovery rate is low in the elderly, and sarcopenia contributes to kyphotic changes after laminoplasty [23, 25].

There are some limitations in our study. First, this was a single-center study with a small sample size. Therefore, despite the fact that we consecutively selected our patients, the results may not be generalizable. Second, our study was a retrospective study, and unintended biases including selection bias and information bias could be present. Furthermore, we did not consider surgical procedures such as single-door laminoplasty or double-door laminoplasty, either or both could be related to postlaminoplasty deformity. Third, our follow-up period was 1 year, which was chosen for reasons such as loss to follow-up; more important changes may emerge if longer follow-up is considered. To our knowledge, there are only few studies comparing cervical sagittal alignment after cervical laminoplasty of older and younger patients. Further studies may validate our findings.

## 5. Conclusions

There are more significant postoperative changes of C2-7 SVA after cervical laminoplasty in elderly patients than in younger patients. When planning cervical laminoplasty in elderly patients, careful attention should be paid.

## Figures and Tables

**Table 1:** Preoperative range, mean value, and standard deviation of each parameter in elderly patients (*N* = 30) vs. young patients (*N* = 30).

	Elderly patients (*N* = 30)			Young patients (*N* = 30)		
	Minimum	Maximum	Mean (SD)	Minimum	Maximum	Mean (SD)
Age	65.00	84.00	70.80 (5.03)	36.00	64.00	52.53 (7.68)
C2-7 SVA	-0.60	5.10	2.09 (1.26)	0.70	4.80	1.97 (0.86)
CL	2.70	31.60	13.98 (7.86)	5.00	33.00	12.59 (6.47)
T1 slope	4.90	48.60	26.16 (9.73)	15.00	46.00	25.43 (6.34)
T1 slope-CL	2.10	27.10	13.22 (5.83)	-1.00	29.50	12.84 (5.89)

In the older groups (men, *n* = 18), the mean age was 70.8 yrs. The preoperative data were as follows: C2-7 SVA mean range, 2.09; CL mean range, 13.98; T1 slope mean range, 26.16; and T1 slope-CL mean range, 13.22. In the younger group (men, *n* = 20), the mean age was 52.53 yrs. The preoperative data were as follows: C2-7 SVA mean range, 1.97; CL mean range, 12.59; T1 slope mean range, 25.43; and T1 slope-CL mean range, 12.84. ^∗^*C2-7 SVA*: C2-7 sagittal vertical axis; *CL*: cervical lordosis; *T1 slope-CL*: T1 slope minus cervical lordosis.

**Table 2:** Change in sagittal alignment parameters in patients after laminoplasty (1-year follow-up).

	Elderly group (30 cases)				Young age group (30 cases)			
	Preoperative	Postoperative	*∆*	*P*	Preoperative	Postoperative	*∆*	*P*
C2-7 SVA	2.09	3.39	1.30	<0.01^∗^	2.31	2.94	0.63	<0.01∗
CL	13.98	13.04	-0.94	0.402	12.59	14.03	1.44	0.234
T1 slope	26.16	28.84	2.68	0.075	25.43	28.10	2.66	<0.01^∗^
T1 slope-CL	13.22	17.19	3.97	0.017∗	12.91	14.73	1.81	0.112

The postoperative change of C2-7 SVA tended to be greater than preoperative values in both age groups. In the older group, C2-7 SVA and T1 slope-CL were significantly changed postoperatively (*P* < 0.01 and *P* = 0.017, respectively). In the younger group, C2-7 SVA and T1 slope were significantly changed (*P* < 0.01 and *P* < 0.01). ^∗^*C2-7 SVA*: C2-7 sagittal vertical axis; *CL*: cervical lordosis; *T1 slope-CL*: T1 slope minus cervical lordosis. *∆* = Postoperative parameter value–preoperative parameter value. *P* < 0.05, statistically significant.

**Table 3:** Change in sagittal alignment parameters in patients after laminoplasty (1-year follow-up).

	All patients (60 cases)			
	Preoperative	Postoperative	*∆*	*P*
C2-7 SVA	2.20	3.16	0.96	<0.01^∗^
CL	13.28	13.54	0.25	0.758
T1 slope	25.79	28.47	2.68	<0.01^∗^
T1 slope-CL	13.06	15.96	2.90	<0.01^∗^

C2-7 SVA: C2-7 sagittal vertical axis; CL: cervical lordosis; T1 slope-CL: T1 slope minus cervical lordosis. *∆* = Postoperative parameter value–preoperative parameter value. ^∗^*P* < 0.05, statistically significant.

**Table 4:** Mann-Whitney *U* test of sagittal alignment parameter changes in patients after laminoplasty.

	Mean rank	Sum of rank	*P* value
*∆*C2-7 SVA			
Elderly group (*N* = 20)	38.63	1159.00	<0.01^∗^
Young age group (*N* = 20)	22.37	671.00	
*∆*T1 slope			
Elderly group (*N* = 20)	31.27	938.00	0.734
Young age group (*N* = 20)	29.73	892.00	
*∆*CL			
Elderly group (*N* = 20)	27.55	826.50	0.191
Young age group (*N* = 20)	33.45	1003.50	
*∆*T1 slope-CL			
Elderly group (*N* = 20)	27.22	816.50	0.145
Young age group (*N* = 20)	33.78	1013.50	

*C2-7 SVA*: C2-7 sagittal vertical axis; *CL*: cervical lordosis; *T1 slope-CL*: T1 slope minus cervical lordosis. *∆* = Postoperative parameter value–preoperative parameter value. ^∗^*P* < 0.05, statistically significant.

## Data Availability

The [SPSS] data used to support the findings of this study have been deposited in the [Figshare] repository (DOI: 10.6084/m9.figshare.12860978). The [SPSS] data used to support the findings of this study are included within the article. The [SPSS] data used to support the findings of this study are included within the supplementary information file(s). The [SPSS] data used to support the findings of this study were supplied by [Figshare] under license and so cannot be made freely available. Requests for access to these data should be made to [Figshare, Cervical sagittal imbalance after cervical laminoplasty in elderly patients]. The [SPSS] data used to support the findings of this study are currently under embargo while the research findings are commercialized. Requests for data, [6 months] after publication of this article, will be considered by the corresponding author. The [SPSS] data used to support the findings of this study may be released upon application to the [Age related laminoplasty or Hyunwoong Mun], who can be contacted at [Cervical sagittal imbalance after cervical laminoplasty in elderly patients]. The [SPSS] data used to support the findings of this study are restricted by the [Hyunwoong Mun] in order to protect [PATIENT PRIVACY]. Data are available from [Hyunwoong Mun, Cervical sagittal imbalance after cervical laminoplasty in elderly patients] for researchers who meet the criteria for access to confidential data. Previously reported [SPSS] data were used to support this study and are available at [DOI: DOI: 10.6084/m9.figshare.12860978]. The [SPSS] data supporting this [SYSTEMATIC REVIEW] are from previously reported studies and datasets, which have been cited. The [SPSS] data used to support the findings of this study are available from the corresponding author upon request. No data were used to support this study.
